# The successful treatment of a Gustilo–Anderson type IIIc distal leg injury with a large bone defect in elderly patient with severe osteoporosis: a case report

**DOI:** 10.1186/s13256-023-04193-5

**Published:** 2023-10-13

**Authors:** Koji Nozaka, Naohisa Miyakoshi, Motoki Mita, Yoichi Shimada

**Affiliations:** Department of Orthopedic Surgery, Akita University Graduate School of Medicine, 1-1-1 Hondo, Akita, 010-8543 Japan

**Keywords:** Gustilo–Anderson type IIIc open tibial fracture, Severe osteoporosis, Ilizarov external fixator

## Abstract

**Background:**

Gustilo–Anderson type IIIc tibial open fracture with large bone defects in elderly patients with severe osteoporosis is a rare injury that may be a challenging clinical scenario.

**Case presentation:**

This study presents the case of a 68-year-old Japanese man who sustained a Gustilo–Anderson type IIIc open tibial fracture with a large bone defect. The patient had severe osteoporosis and the bone was contaminated; therefore, we determined that the bone could not be returned to the tibia. The patient underwent acute limb shortening and gradual lengthening with an Ilizarov external fixator combined with low-intensity pulsed ultrasound and teriparatide administration for limb reconstruction, which allowed immediate full weight-bearing capacity. The fixator was removed at 12 months postoperatively, and by this time, the fracture had completely healed. At the most recent 5-year follow-up after the injury, the patient reported full weight-bearing capacity without walking aids and had full knee and ankle range of motion.

**Conclusions:**

To the best of our knowledge, this is the first study to report the use of combined Ilizarov technique, low-intensity pulsed ultrasound, and teriparatide for limb reconstruction of Gustilo–Anderson type IIIc open tibial fractures with large bone defects in elderly patients with severe osteoporosis.

## Background

The incidence of open tibial fractures in the elderly is increasing due to the growing elderly population. Open tibial fractures with large bone defects are an increasing problem, and their treatment is challenging. Gustilo–Anderson type IIIc open tibial fractures with large bone defects are less common in elderly patients with severe osteoporosis than in young patients. However, the literature on the outcomes of open tibial fractures with large bone defects in elderly patients treated with modern techniques is limited.

## Case presentation

**Fig. 1:**
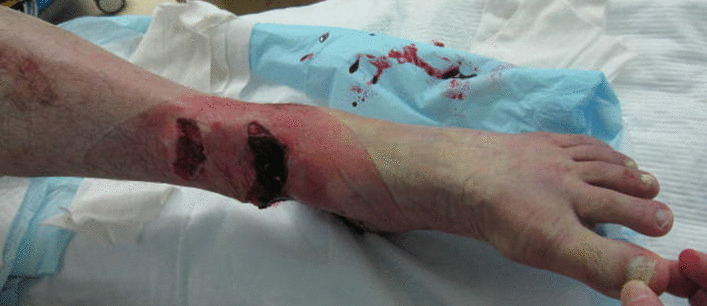
Two large soft-tissue defects in the medial left ankle

**Fig. 2:**
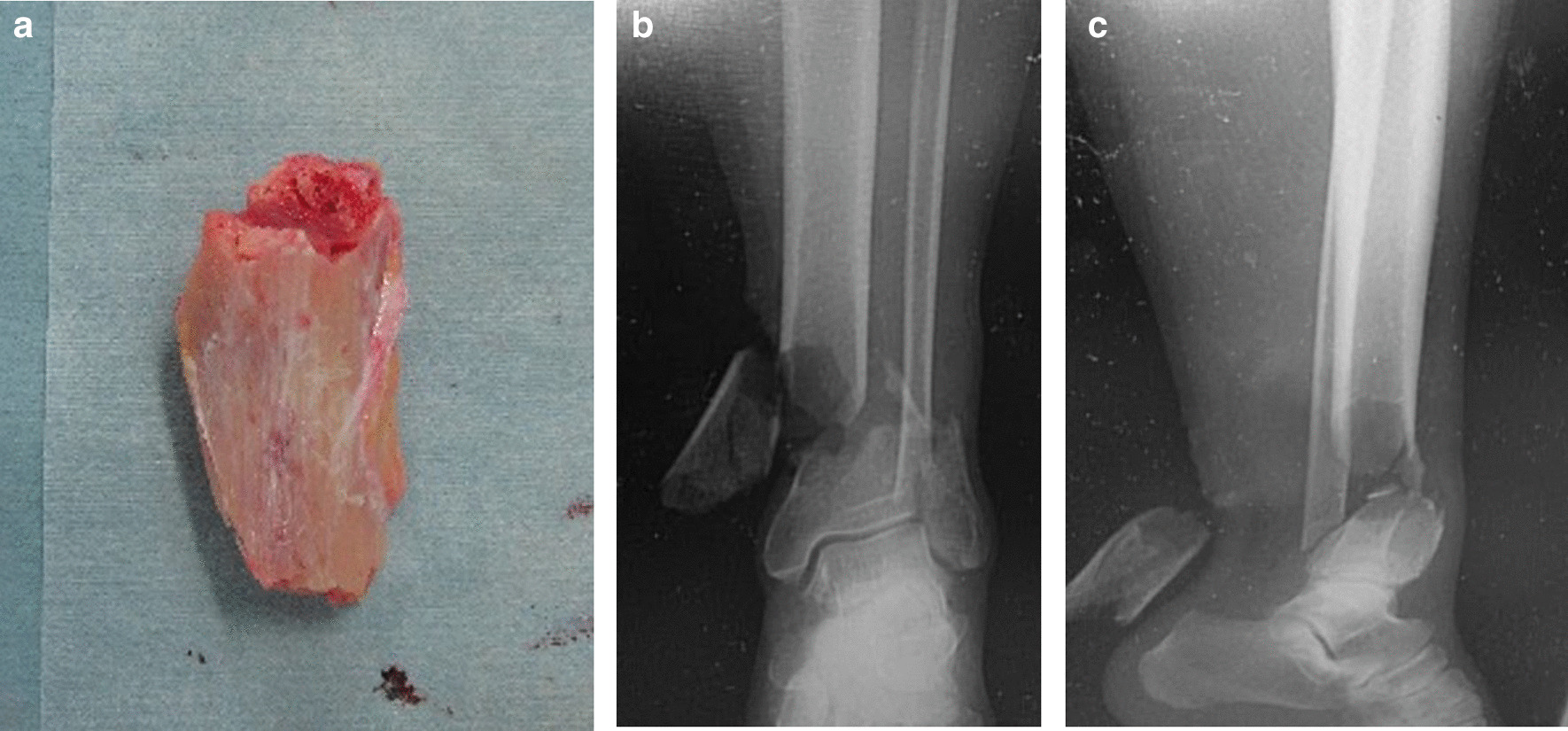
**a** The contaminated large bone that fell out of the body; **b** and **c** X-ray images taken in the emergency room

**Fig. 3:**
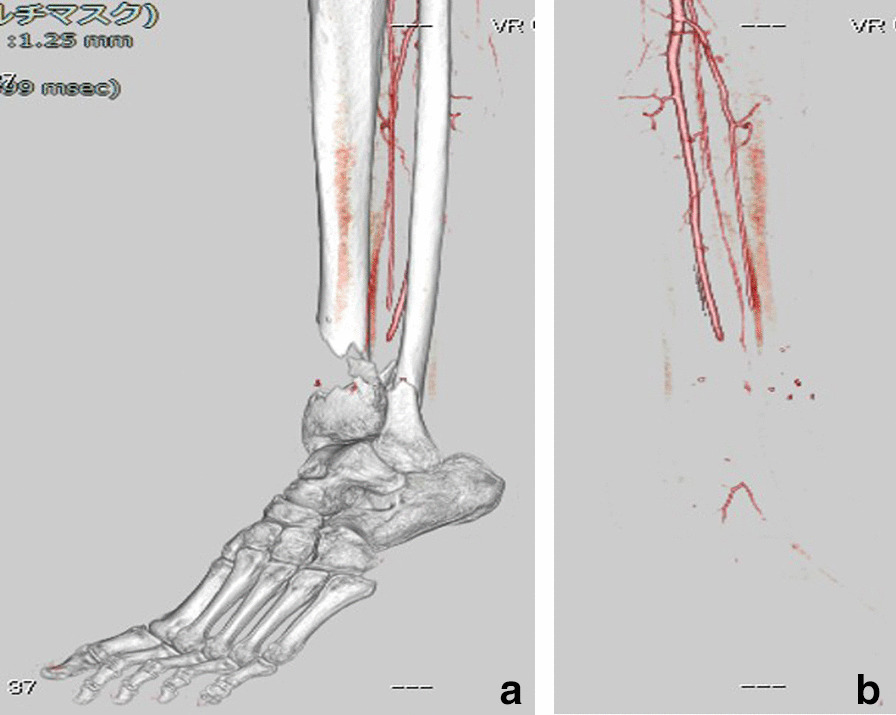
**a** 3D computed tomography angiography image with the bone; **b** 3D computed tomography angiography image without the bone *3D* three-dimensional

**Fig. 4:**
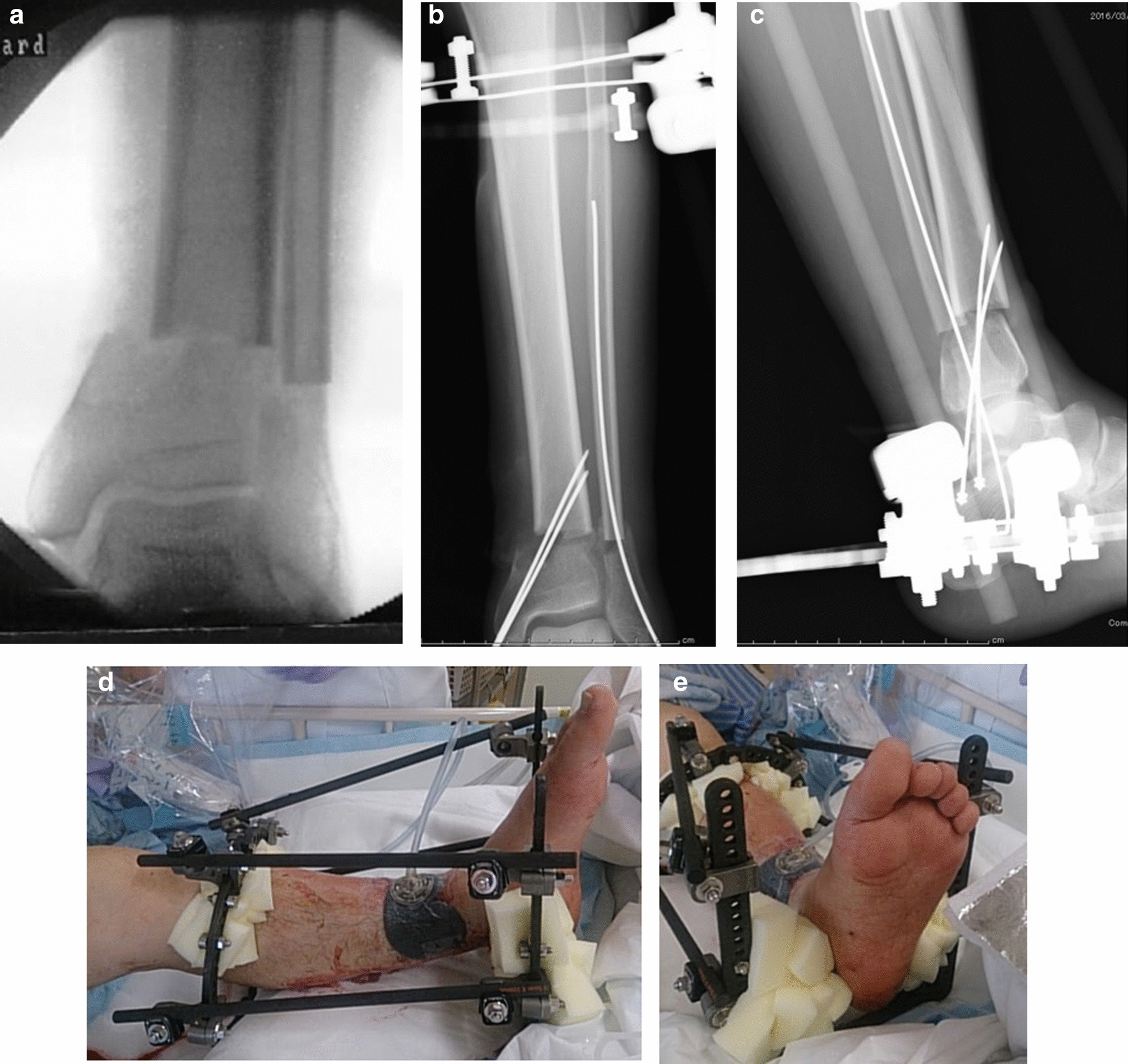
**a** Shortened fibula with a 75-mm osteotomy to match the length of the tibia; **b** and **c** the fibula was fixed with an Ilizarov external fixator for temporary ankle joint-bridging fixation; **d** and **e** clinical photograph of the fibula, which was fixed with an Ilizarov external fixator for temporary ankle joint-bridging fixation

A 68-year-old Japanese man was injured while electrically reeling a wire rope on a fishing boat. Shortly after, the patient was transported to our hospital’s emergency room, where he was treated for hypertension. The patient had no relevant family or psychosocial history. On physical examination, two large soft-tissue defects were observed in the medial left ankle (Fig. 1). The patient’s left foot was pale, and we did not palpate any arteries in his lower legs because of severe ankle pain. Next, pulses from the peroneal, tibialis anterior, and tibialis posterior arteries, as well as the capillary refill, color, and temperature, were compared between the injured and uninjured feet. Then, the paramedics brought in a 75-mm tibia that had fallen out of the body on the fishing boat. The bone was contaminated; therefore, we determined that the bone could not be returned to the tibia (Fig. 2a–c). Three-dimensional (3D) computed tomography (CT) angiography revealed injury to the peroneal, tibialis anterior, and tibialis posterior arteries (Fig. 3a, b). The preoperative Mangled Extremity Severity Score (MESS) was 7 points. We considered limb salvage feasible and performed reconstructive surgery. A total of 6 hours after the injury, we debrided the soft tissues of the injury in the operating room (day 0). After debridement, the soft-tissue defect measured 40 mm × 80 mm. We decided that acute limb shortening and gradual lengthening would be a reasonable method for rapid revascularization. Accordingly, we shortened the fibula with a 75-mm osteotomy to match the length of the tibia. We resected and prepared a spike in the distal tibial fragment (Fig. 4a). We crimped the fracture sufficiently and fixed it with an Ilizarov external fixator for temporary ankle joint-bridging fixation (Fig. 4b, c). We reconstructed the anterior and posterior tibial arteries and veins with end-to-end anastomosis (day 0). No severe nerve injury to the lower leg was observed.

Revascularization was completed 6 hours after the injury, after which circulation in the injured foot improved. The size of the soft-tissue defect was now 20 × 80 mm. Negative pressure wound therapy was used to address the soft-tissue defect (Fig. 4d). After the patient gained consciousness following anesthesia, ankle joint circulation was good and automatic movement of the ankle joint and toes was possible (Fig. 4e). The patient had normal motor and sensory nerves in his lower leg. Although the patient had mild renal dysfunction, he did not undergo hemofiltration and recovered without permanent organ damage.

**Fig. 5:**
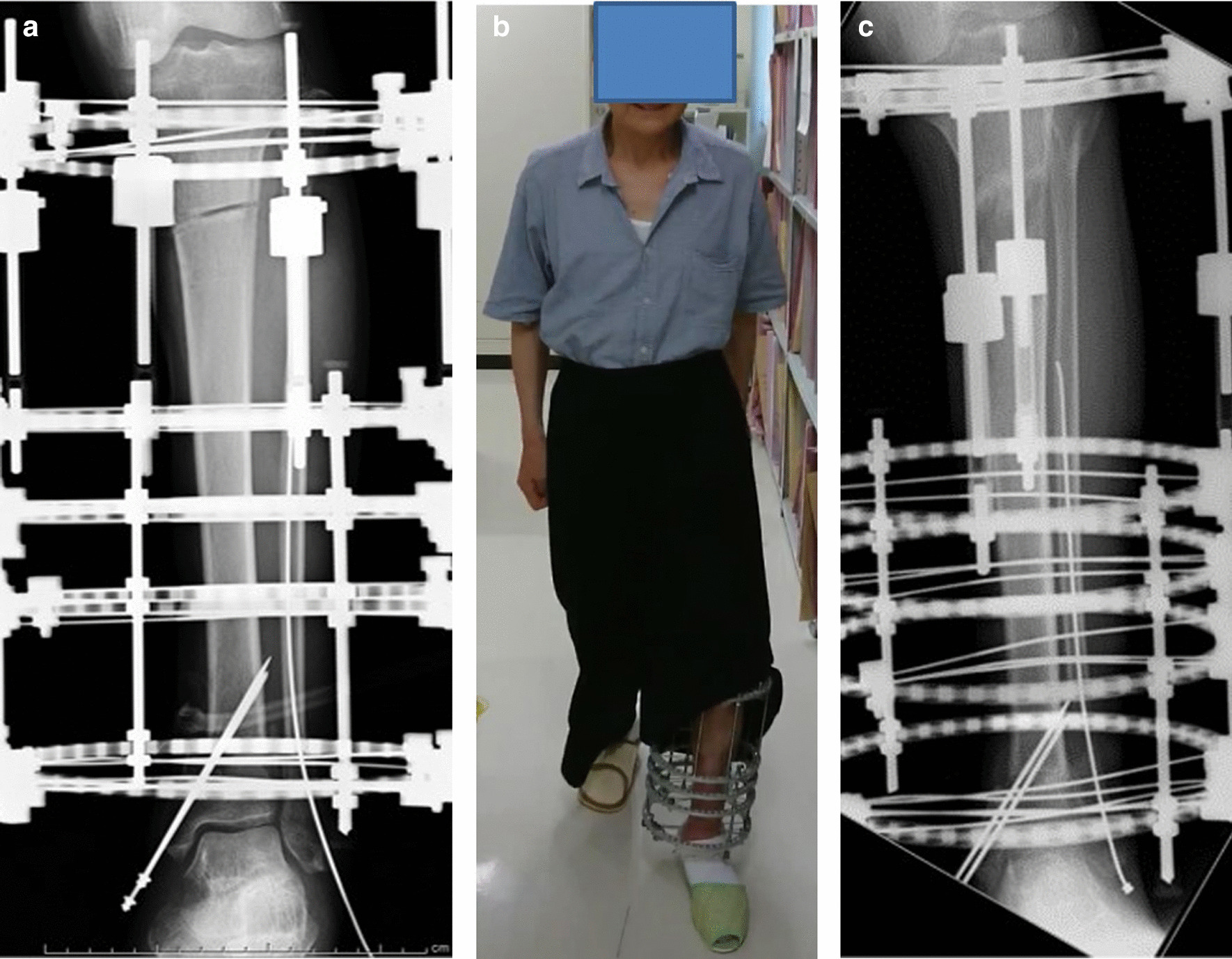
**a** Osteotomy at the proximal tibia with an Ilizarov external fixator for gradual lengthening; **b** bone lengthening at a rate of 0.75 (0.25 × 3) mm/day; **c** walking with full weight-bearing capacity immediately after surgery

A total of 2 days after the injury (day 2), we cleaned the open fracture again, performed additional debridement, and reapplied negative pressure wound therapy. A total of 14 days after the injury (day 14), osteotomy was performed at the proximal tibia with an Ilizarov external fixator for gradual limb lengthening (Fig. 5a). A total of 3 weeks after the injury (day 21), gradual lengthening of the proximal tibia was initiated. We noted that the bone strength was weak at the time of wire insertion; therefore, we measured the bone density and identified primary osteoporosis. A baseline dual-energy X-ray absorptiometry scan showed that the bone mineral density at the femoral neck was 0.441 g/cm^2^ and T-Score was −3.3 standard deviation. He was started on a once-weekly injection of 56.5 μg of teriparatide. Furthermore, after osteotomy, treatment with a low-intensity pulsed ultrasound stimulation (LIPUS) device (SAFHS 2000, Exogen, Inc., Piscataway, NJ) was started for 20 minutes/day at the fracture and osteotomy sites. This device had a frequency of 1.5 MHz, a signal burst width of 200 μs, a signal repetition frequency of 1 kHz, and an intensity of 30 mW/cm^2^. The patient was allowed to walk with full weight-bearing capacity immediately after the surgery (Fig. 5b) (Day 22). He also performed knee and ankle range of motion exercises (day 22). We performed bone lengthening at a rate of 0.75 (0.25 × 3) mm/day. The patient continued to undergo bone lengthening at home after discharge. The patient visited the outpatient clinic once a month for radiography. The patient was positive about returning to his original job, and was consistently mentally stable. Radiography performed 1 year later revealed good callus formation (Fig. 5c). The fixator was removed after one year. The patient returned to his original job as a fisherman 2 months after the fixator removal. The patient’s 14 months of treatment was covered by industrial accident compensation insurance.

**Fig. 6:**
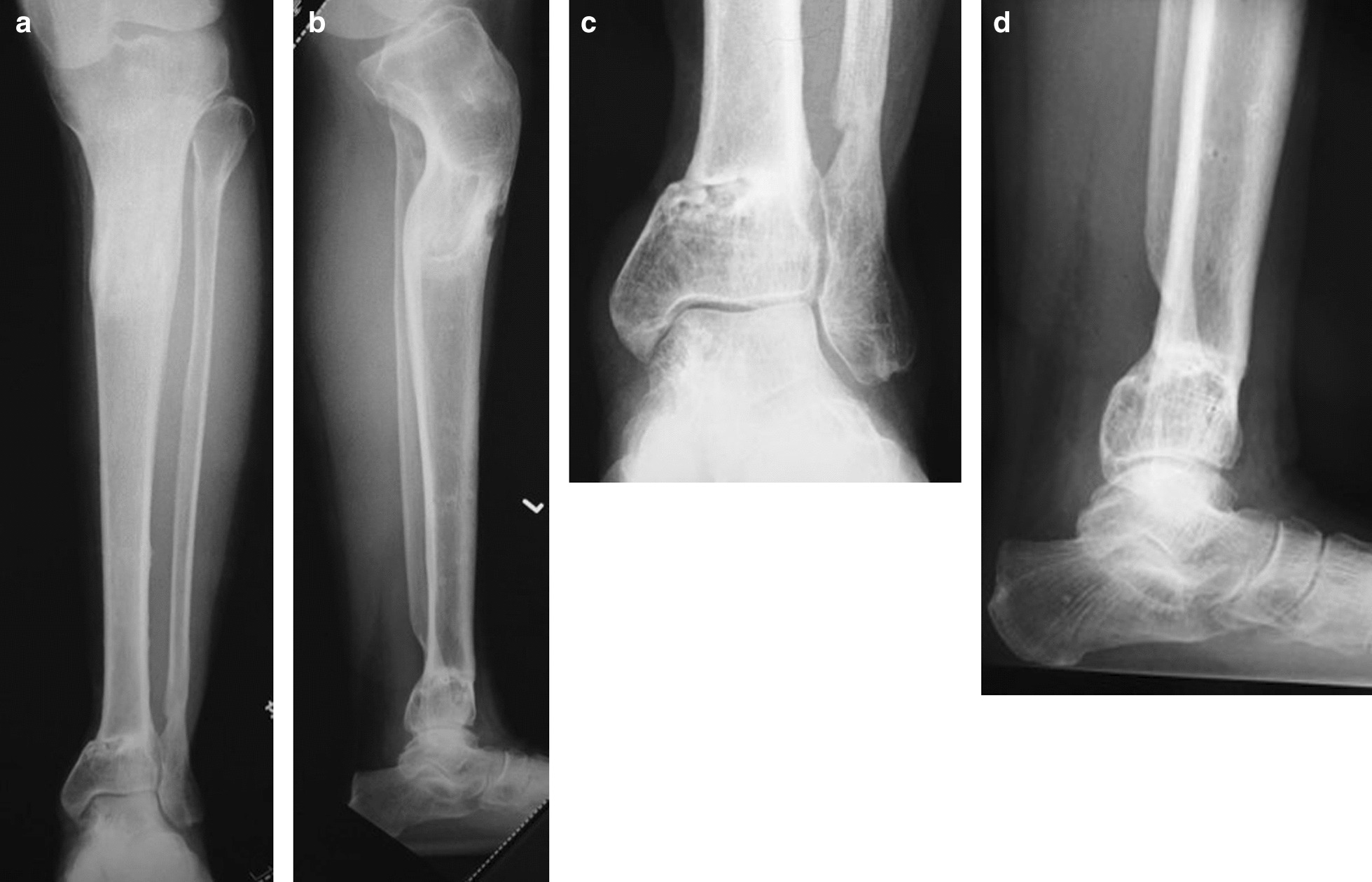
**a**–**d** X-ray images taken at postoperative 5-year follow-up

**Fig. 7:**
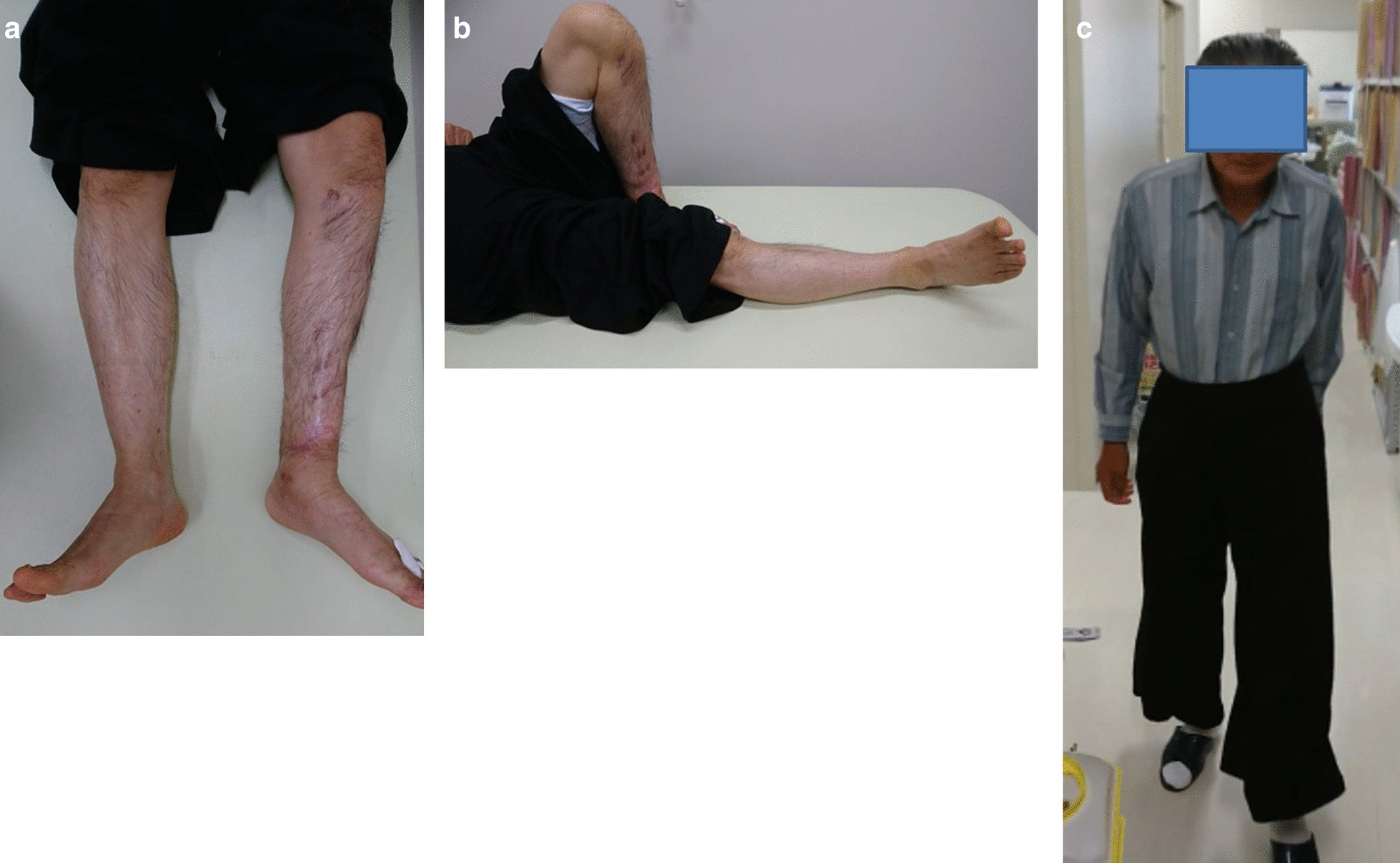
**a**–**c** Clinical photograph at postoperative 5-year follow-up

At the 5-year follow-up after injury, radiographs showed good callus formation and bone union (Fig. 6a, b, c, and d). The patient was independently mobile, with a knee range of motion of 0–140°, ankle dorsiflexion of 5°, and plantar flexion of 50° (Fig. 7a, b, and c). During the 12 months in which the fixator was inserted, there were a few superficial pin-tract infections, which were treated with empirical oral antibiotics and daily pin-tract dressings. A total of 5 years after surgery, the patient returned to work without any problems, with an American Orthopaedic Foot and Ankle Society (AOFAS) score of 95 points. The clinical results, according to the AOFAS score, were excellent in this patient.

## Discussion and conclusions

Treatment of high-energy open tibial fractures is challenging. Gustilo–Anderson type IIIc open tibial fractures were mainly treated with amputation in the past [1]. The complication rates are high for Gustilo–Anderson type IIIc open tibial fractures, which are associated with more severe soft-tissue loss and/or arterial injury requiring repair [2]; these complications include secondary amputation, nonunion, infection, and malunion [3–5]. The acceptance rate of amputation is poor in eastern-culture patients, especially in Japanese elderly patients [6]. Adaptation to a prosthesis is relatively easier in young amputees than in elderly amputees [7]. In our patient, there was a bone defect, soft-tissue defect, and peroneal, tibialis anterior, and tibialis posterior artery tears. The advantage of acute shortening is that it is easier to reconstruct the arteries and veins with end-to-end anastomosis. Furthermore, soft-tissue defects were smaller in genaral; therefore, function reconstruction was possible in our elderly patient, despite his presentation. In Gustilo–Anderson type IIIb open tibial fractures treated with acute shortening, gradual lengthening is usually subjected to a second-stage procedure performed after union of the fracture [8]. The main disadvantage of this approach is prolonged treatment duration. In Gustilo–Anderson type IIIc open tibial fractures, lengthening is delayed to prevent traction on vascular anastomoses as they heal, which may potentially risk the revascularization procedure. In our study, we performed early lengthening without causing any harm to vascular repair, as lengthening was performed at the proximal tibia most distant from the vascular injury. Management of open injuries of the limbs are challenging, as there are still many gray areas in decision making regarding salvage, timing, and reconstruction type. As a result, there is still an unacceptable rate of secondary amputations, which leads to tremendous waste of resources and psychological devastation of the patient and their family [9]. In addition, limb salvage is more cost effective than amputation and prosthesis use [10, 11]. Our patient achieved a satisfactory functional status and avoided psychological trauma due to amputation; he was able to return to his original position as a fisherman.

It is difficult to reconstruct large bone defects in elderly patients with severe osteoporosis due to decreased bone formation. LIPUS has been used to treat leg lengthening [12, 13]. Intermittent administration of human parathyroid hormone (PTH) has an anabolic effect on the bone in humans and is expected to be a potent agent for fracture healing [14]. Several recent studies have revealed that intermittent treatment with PTH stimulates osteogenesis in experimental fracture healing of cortical bones and that the effects of PTH on cortical bone repair are site-specific. Aspenberg *et al*., in a prospective randomized double-blind study of conservative fracture treatment for 102 postmenopausal women with distal radial fractures, showed that the time to healing was shorter in patients who received 20 mg teriparatide than in the placebo group patients [15]. Warden *et al*. reported that teriparatide and LIPUS have contrasting additive rather than synergistic effects during fracture healing [16]. Teriparatide primarily increased the callus bone mineral content without influencing its size, whereas LIPUS increased callus size without influencing the callus bone mineral content in rat models [16]. We have reported combined effect of teriparatide and low-intensity pulsed ultrasound for patients with nonunion [17]. Furthermore, we have reported intractable fractures such as pathological fractures in patients with Alagille syndrome or nonunion after ankle fracture for Charcot arthropathy that was treated with LIPUS and an Ilizarov external fixator [18, 19]. Early ambulation and immediate weight-bearing capacity may improve limb circulation and enhance the healing process, based on the fact that the speed of fracture healing is usually proportional to the amount of available circulation to and between fragments [20, 21].

One of the most important advantages of using Ilizarov external fixators is the excellent recorded knee and ankle range of motion within a short time after surgery. Active and passive movements of both the joints were allowed and encouraged during the entire course of treatment immediately after application of the frame. The main disadvantages of Ilizarov external fixators are that they are technically demanding and there is absolute necessity of adequate care of the frame. From our point of view, the fact that the patient could achieve immediate weight-bearing capacity and could be discharged and returned to work is adequate justification for this procedure.

## Data Availability

The datasets used and/or analyzed during the current study are available from the corresponding author on reasonable request.

## Abbreviations

LIPUS: Low-intensity pulsed ultrasound stimulation
PTH: Parathyroid hormone
